# Evaluating the cost-consequence of a standardized strategy for the etiological diagnosis of uveitis (ULISSE study)

**DOI:** 10.1371/journal.pone.0228918

**Published:** 2020-02-14

**Authors:** Audrey de Parisot, Yvan Jamilloux, Laurent Kodjikian, Marie-Hélène Errera, Neila Sedira, Emmanuel Heron, Laurent Pérard, Pierre-Loïc Cornut, Christelle Schneider, Sophie Rivière, Priscille Ollé, Grégory Pugnet, Pascal Cathébras, Pierre Manoli, Bahram Bodaghi, David Saadoun, Stéphanie Baillif, Nathalie Tieulie, Marc André, Frédéric Chiambaretta, Nicolas Bonin, Philip Bielefeld, Alain Bron, Frédéric Mouriaux, Boris Bienvenu, Nassira Amamra, Pascale Guerre, Evelyne Decullier, Pascal Sève, on behalf of the ULISSE group

**Affiliations:** 1 Department of Internal Medicine, Hospices Civils de Lyon, Croix-Rousse Hospital, Lyon, France; 2 Department of Ophthalmology, Hospices Civils de Lyon, Croix-Rousse Hospital, Lyon, France; 3 Department of Ophthalmology, Quinze-Vingts Hospital, Paris, France; 4 Department of Internal Medicine, Quinze-Vingts Hospital, Paris, France; 5 Department of Internal Medicine, Edouard-Herriot Hospital, Lyon, France; 6 Department of Ophthalmology, Edouard-Herriot Hospital, Lyon, France; 7 Department of Ophthalmology, Centre Hospitalier Regional Universitaire de Montpellier, Montpellier, France; 8 Department of Internal Medicine, Centre Hospitalier Regional Universitaire de Montpellier, Montpellier, France; 9 Department of Ophthalmology, Pierre-Paul Riquet Hospital, Toulouse, France; 10 Department of Internal Medicine, Purpan University Hospital, Toulouse, France; 11 Department of Internal Medicine, Centre Hospitalier Universitaire de Saint-Etienne, Saint-Étienne, France; 12 Department of Ophthalmology, Centre Hospitalier Universitaire de Saint-Etienne, Saint-Étienne, France; 13 Department of Ophthalmology, Pitié-Salpêtrière Hospital, Paris, France; 14 Department of Internal Medicine, Pitié-Salpêtrière Hospital, Paris, France; 15 Department of Ophthalmology, Centre Hospitalier Universitaire de Nice, Nice, France; 16 Department of Internal Medicine, Centre Hospitalier Universitaire de Nice, Nice, France; 17 Department of Internal Medicine, Gabriel-Montpied Hospital, Clermont-Ferrand, France; 18 Department of Ophthalmology, Gabriel-Montpied Hospital, Clermont-Ferrand, France; 19 Department of Internal Medicine, Centre Hospitalier Universitaire de Dijon, Dijon, France; 20 Department of Ophthalmology, Centre Hospitalier Universitaire de Dijon, Dijon, France; 21 Department of Ophthalmology, Centre Hospitalier Universitaire de Caen, Caen, France; 22 Department of Internal Medicine, Centre Hospitalier Universitaire de Caen, Caen, France; 23 Pole IMER, Hospices Civils de Lyon, Lyon, France; Universita degli Studi di Firenze, ITALY

## Abstract

**Main objective:**

To prospectively assess the cost-consequence of a standardized diagnostic strategy as to compared to an open one for the etiological diagnosis of uveitis.

**Design:**

This was a prospective, non-inferiority, multicentre, randomized controlled trial.

**Methods:**

We included all consecutive patients with uveitis who had visited at least one of the Departments of Ophthalmology. In the standardized group, patients had a minimal work-up regardless of the type of uveitis (including evaluation of the CBC, ESR, C-reactive protein, tuberculin skin test, syphilis serology and chest X-ray). Depending on ophthalmological findings, further investigations could be performed. In the open strategy, ophthalmologists were free to order any kind of investigation. The main outcome was the mean cost per patient of each strategy.

**Results:**

903 uveitis patients were included from January, 2010 to May, 2013. The mean cost per patient of the standardized strategy was 182.97 euros [CI 95% (173.14; 192.80)], and the mean cost per patient of the open strategy was 251.75 euros [CI 95% (229.24; 274.25)]. Therefore, the mean cost per patient of the standardized strategy was significantly lower than the mean cost per patient of the open strategy (p<0.001). There were significantly fewer visits (p<0.001), fewer radiological procedures (p<0.004) and fewer laboratory investigations (p<0.001) in the standardized group.

**Conclusion:**

A standardized strategy is a cost-saving approach for the etiological diagnosis of uveitis.

## Introduction

Uveitis, which can be defined as an inflammation of the uveal tract, can be caused by many infectious and non-infectious disorders such as systemic diseases, ocular specific disorders or may be drug-induced. However, it remains idiopathic in 25–45% of the cases [1–7] in Western countries. Uveitis accounts for approximately 5–10% of preventable blindness in the US and up to 15% worldwide [8–11], which is why it is important to search for a specific aetiology in order to start an appropriate treatment. The etiological diagnosis of uveitis remains a challenge due to the wide variety of diagnoses. An accurate history and detailed physical examination are the first steps in evaluating a patient with uveitis [12]. Then, on the basis of clinical findings, the physician may order various diagnostic tests. However, a non-selective approach to testing can be very costly, and is not always efficient, since many tests have a low diagnostic yield [13]. For example, a Canadian study showed that ophthalmologists ordered more diagnostic tests than those recommended by evidence-based guidelines for investigating anterior uveitis, including tests with low diagnostic yields. When applied to the Canadian population, this was responsible for an additional cost of 600,000 dollars/year to the Canadian health care system [14,15]. Physicians might have a broad approach leading to unnecessary investigations for fear of missing a diagnosis. However, performing many tests, which are not supported by clinical, or paraclinical findings, may lead to misinterpretation of false positive results and unnecessary supplementary investigations or treatments.

In the ULISSE study, a controlled trial that has evaluated the benefits of a standardized strategy for the etiologic diagnosis of uveitis [16], we prospectively assessed the costs of a standardized approach, in which all patients had a minimal work-up regardless of the type of uveitis (CBC, ESR, C-reactive protein, tuberculin skin test, syphilis serology, and chest X-ray) followed by more complex investigations, ordered by ophthalmologists, if needed. This standardized strategy was compared to an open one in which ophthalmologists could order any kind of investigation. In this study, the standardized strategy appeared to be a cost-saving diagnostic approach for the etiological diagnosis of uveitis.

Economic evaluations are useful to assess the cost of current practice patterns, and to determine the potential cost savings of establishing new approaches. Unfortunately, there are few studies evaluating the diagnostic yield of investigations and the cost-consequence of a strategy for the etiological diagnosis of uveitis.

Therefore, the main aim of this study was to assess the cost-consequence of the standardized diagnostic approach evaluated in the ULISSE study, compared to the open strategy.

## Material and methods

### Ethics

The ULISSE study was approved by a French institutional review board (*Comité de Protection des Personnes Sud-Est IV*). It was conducted in accordance with the Declaration of Helsinki. Patients included in the study have provided their written informed consent. The ULISSE study is registered under the unique ID #NCT01162070 at www.clinicaltrials.gov.

### Design

The study design has been reported in detail previously [16]. Briefly, it was a multicentre, non-inferiority, prospective, randomized controlled trial evaluating two strategies for the etiological diagnosis of uveitis; an open strategy vs. a standardized one. In the open strategy, ophthalmologists were free to order any investigation and to refer the patient to the internal medicine department. Conversely, in the standardized strategy, regardless of the type of uveitis, a minimal work-up was performed after careful examination of the patient by both the ophthalmologist and the internist. Then, depending on clinical or paraclinical findings, extra diagnostic tests could be ordered. When no diagnosis was done at the end of the standardized strategy, physicians could perform free investigations. However, such a result was considered as a failure of the standardized strategy.

In the present study, we compared the cost-consequence of both strategies.

### Patients

Inclusion and exclusion criteria have been reported previously [16]. Briefly, we included consecutive patients, who visited at least one of the participating departments of ophthalmology, for a diagnosis of uveitis, between June, 2010 and May, 2013. The diagnosis of uveitis was always established after careful ophthalmological examination and the anatomical localization was classified according the Standardization of Uveitis Nomenclature [17]. Ophthalmologists or internists had to retain an etiological diagnosis at month 6 whenever possible. In the absence of a diagnosis, the internist had to perform a new examination of the patient at month 12 to look for new signs or symptoms (except when uveitis was an acute anterior one).

### Outcomes

The primary outcome was the mean cost per patient of the standardized strategy as compared to the open one. Secondary outcomes were the mean cost per patient of each step and the cost of extra free investigations.

### Evaluation of costs

To evaluate the direct costs of each diagnostic strategy, we first estimated the cost of diagnostic tests (laboratory, radiological, endoscopic, and microsurgery procedures), as well as the cost of visits to specialists. The costs of laboratory investigations were estimated with the current tariffs of the National Biology Table (accessed at http://www.codage.ext.cnamts.fr/codif/nabm/). The costs of radiological, endoscopic, and microsurgery procedures were evaluated with the current tariffs of the Common Classification of Medical Acts and the costs of visits to specialists were evaluated with the current tariffs of the general classification system for professional activities. The costs of antibiotic treatments, hospitalizations and work stoppages were not estimated in the analyses:

- Data on antibiotic treatments were not sufficient to allow their evaluation. We had no information on the dose prescribed, the dose administered, or the treatment duration.
- To estimate the costs of hospitalizations, all the medical information departments of the participating institutions were solicited (n = 23), but only eight establishments responded. This did not allow us to estimate the hospitalization costs of the patients included in the ULISSE study.
- With regards to work stoppages, no data was reported.

### Statistical analysis

We performed non-parametric tests with Fischer exact test on costs data. Then, we wanted to test the mean annual costs per patient. So we compared the means between groups with a Student t-test, because the number of patient was sufficient. All analyses were performed using a IBM SPSS statistics version 19.

### Data sharing statement

All data are available on request. Due to the date of study conception (2010), patients of the present study have not been informed and therefore have not consented that their data could be publicly shared.

## Results

**Fig 1:**
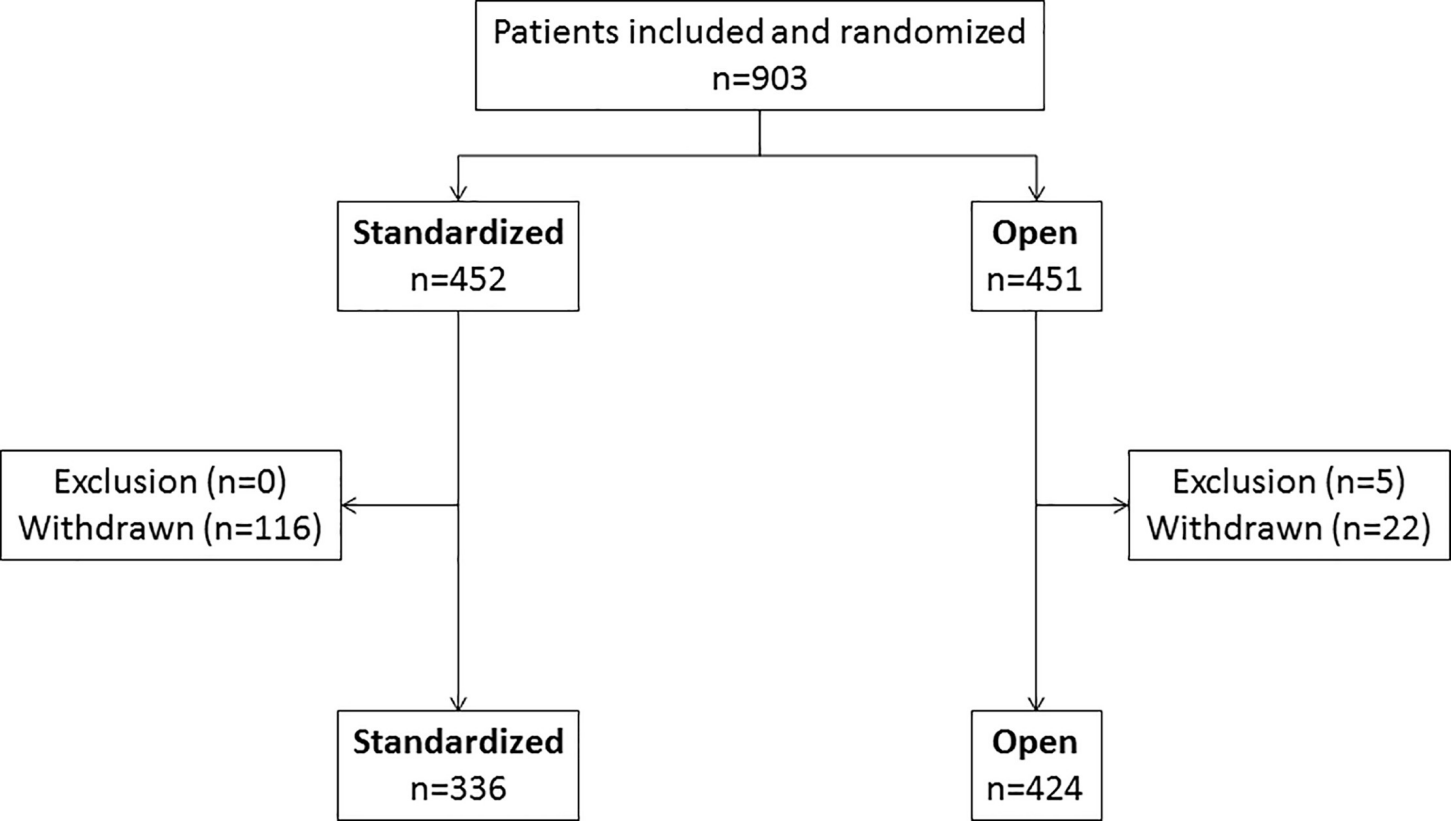
Study flow chart.

Over the study period, 903 patients with uveitis were included and randomized. Five patients were excluded and 138 were withdrawn, therefore there were 336 patients in the standardized strategy and 424 in the open strategy (Fig 1). In the standardized group, 25.6% of the patients were lost to follow-up [16].

In the standardized strategy, the mean cost per patient of the first step’s investigations was 43.86 euros [95% CI (43.62; 44.09)]. The chest X-ray accounted for 56% of the overall cost for this step, the complete blood count for 22%, the syphilis serology for 12%, and the C-reactive protein/erythrocyte sedimentation rate for 10%.

Among the 151 patients who underwent the second step’s investigations, the mean cost per patient was 60.62 euros [95% CI (56.65; 64.60)]. The HLA-B27 determination accounted for 74.7% of the overall cost, the chest computed tomography for 13%, and the angiotensin-converting enzyme for 6.6%.

Finally, among the 65 patients who underwent the third step’s investigations, the mean cost per patient was 42.14 euros [95% CI (31.48; 52.80)]. The 18F-fluorodeoxyglucose positron emission tomography accounted for 51% of the overall cost, the salivary gland biopsy for 25%, and the lumbar puncture for 12%.

In addition, in the standardized strategy, 335 patients had investigations guided by clinical or paraclinical findings. The mean cost per patient of these investigations was 72.23 euros [95% CI (63.48; 80.99)].

Furthermore, 59 patients had extra free investigations, with a mean cost per patient of 64.51 euros [95% CI (48.05; 80.97)].

Therefore, the mean cost per patient of the standardized strategy was 182.97 euros [CI 95% (173.14; 192.80)]. Visits accounted for 26.84% of the overall cost, radiological procedures for 20.85%, laboratory investigations for 42.75%, and other investigations for 9.56%.

The mean cost per patient of the open strategy was 251.75 euros [CI 95% (229.24; 274.25)]. Visits accounted for 12.62% of the overall cost, radiological procedures for 11.60%, laboratory investigations for 68.37%, and other investigations for 7.41% (Table 1). More specifically, HLA determination accounted for 43% of the overall cost, radiological procedures for 18%, microbiology for 14%, biochemistry for 10%, immunological tests for 9%, and invasive procedures (anterior chamber tap, vitrectomy and lumbar puncture) for 6%.

**Table 1:** Frequently ordered investigations in the open group (>20%). Values are n (%).

**Laboratory investigations**	
Complete blood count	337 (90.35)
Erythrocyte sedimentation rate	322 (86.33)
Serum electrolytes	319 (85.52)
Syphilis serology	308 (82.57)
C-reactive protein	305 (81.77)
Angiotensin converting enzyme	286 (76.68)
Electrophoresis of serum proteins	254 (68.10)
Serum calcium	250 (67.02)
Tuberculin skin test	240 (64.34)
Hepatic tests	200 (53.62)
Antinuclear antibodies	159 (42.63)
IGRA	152 (40.48)
HLA B27	143 (38.34)
Lysozyme	137 (36.73)
Lyme disease serology	132 (35.39)
HLA determination	113 (30.29)
Hepatitis C serology	105 (28.15)
Antineutrophil cytoplamic antibodies	102 (27.35)
Rheumatoid factor	100 (26.81)
Hepatitis B serology	99 (26.54)
Herpes simplex virus 1 and 2 serology	93 (24.93)
Creatinine	93 (24.93)
**Radiological procedures**	
Chest CT	174 (46.65)
Chest X-ray	156 (41.82)
Brain MRI	80 (21.45)

Among the 760 patients included in this study, the mean cost per patient varied depending on the type of uveitis: 268.06€ for posterior uveitis (227.95€ for diagnostic tests and 40.11€ for diagnostic visits), 254.35€ for intermediate uveitis (212.11€ for diagnostic tests and 42.24€ for diagnostic visits), 252.27€ for panuveitis (210.98€ for diagnostic tests and 41.29€ for diagnostic visits) and 198.84€ for anterior uveitis (160.51€ for diagnostic tests and 38.33€ for diagnostic visits) (Table 2). The cost of diagnostic tests for granulomatous and non-granulomatous anterior uveitis were 167.33 € and 158.59€ respectively. There were no statistical differences between both groups except for anterior uveitis: the total cost per patient was significantly lower in the standardized group (163.73 euros vs 233.08 euros, p<0,001) (Table 3).

**Table 2:** Mean cost per patient according to type of uveitis.

	Anterior (n = 478)	Intermediate (n = 58)	Posterior (n = 89)	Panuveitis (n = 128)
Visits	38.33€	42.24€	40.11€	41.29€
Diagnostic tests	160.51€	212.11€	227.95€	210.98€
Total	198.84€	254.35€	268.06€	252.27€

**Table 3:** Mean cost per patient according to type of uveitis in each group.

	Standardized	Open	p-value
Anterior	163.73€	233.08€	<0.001
Intermediate	232.88€	266.53€	0.599
Posterior	232.83€	281.07€	0.560
Panuveitis	221.55€	273.98€	0.218

There were fewer investigations in the standardized strategy than in the open one (3759 vs 5371, p<0.001), with a mean of 12.41 investigations per patient in the standardized strategy versus 15.39 in the open one (p<0.001). There were significantly fewer visits (p<0.001), fewer radiological procedures (p<0.004) and fewer laboratory investigations (p<0.001) in the standardized group. The mean cost per patient of the standardized strategy was significantly lower than the mean cost per patient of the open strategy (p<0.001)

## Discussion

Here, we report a prospective study that assesses the cost-consequence of using a standardized strategy during the etiological work-up for uveitis. The mean cost per patient was 182.97 euros in the standardized strategy [CI 95% (173.14; 192.80)] and 251.75 euros in the open strategy [CI 95% (229.24; 274.25); p<0.001]. Overall, the mean cost per patient is significantly lower in the standardized strategy, and there were significantly fewer investigations in this group. In addition, the mean costs of visits, imaging tests, and laboratory investigations were significantly different between both groups.

There are few medico-economic studies on uveitis. Some studies have evaluated direct costs (hospitalizations, visits, prescription drug use) and indirect costs (disability days) of non-infectious uveitis [18]. However, almost none have evaluated the cost related to diagnostic procedures.

Only Adan-Civera et al. [19] showed that the total cost per patient of non-infectious uveitis in Spain in 2011 ranged from 2811.17 euros for acute anterior uveitis to 18 922.35 euros for posterior uveitis. Diagnostic visits accounted for 9.3% of the overall cost and diagnostic tests for 2.86%. The cost of diagnostic tests and visits in adults varied depending on the type of uveitis: panuveitis and posterior uveitis were the most costly (983€ per patient for visits and 395€ for diagnostic tests) followed by intermediate uveitis (983€ for visits and 303€ for diagnostic tests), acute anterior uveitis (655€ for visits and 156.21€ for diagnostic tests) and chronic anterior uveitis (655€ for visits and 121.89€ for diagnostic tests). In our study, we also found that the cost of diagnostic tests varied depending on the type of uveitis (anterior uveitis being the less costly) but this was not true for the cost of diagnostic visits. In addition, although the cost of diagnostic tests for anterior uveitis was similar in our study, the cost of diagnostic tests for the other types of uveitis was lower in our study. Finally, the cost of visits was much lower in our study.

More recently, Lee et al. reported a web-based survey on 13 patient scenarios in order to examine the range of practice in laboratory testing [13]. The patterns of test utilization were studied and the cost of the testing was calculated based on Noridian Medicare reimbursable rates for Seattle. Eighty-six per cent (12/14) members of the American Uveitis Society executive committee and trustees answered the survey. A total of 45 different tests (laboratory tests, imagings and/or diagnostic procedures) were ordered. The mean number of tests ordered was 5.47 ±2.71 per scenario and per provider whereas in our study there was an average of 12.41 investigations per patient in the standardized group and 15.39 in the open one. The average cost of testing was $282.80 per scenario per provider. In our study, the mean cost of diagnostic tests per patient in the open group was similar (219.97€) but the mean cost in the standardized group was lower (133.86€). In Lee’s study, imaging tests (fluorescein angiography, MRI, chest X-ray, and chest CT) contributed for 59.9% of the total costs of tests, whereas in our study, imaging tests contributed for 13.3 and 28.5% of the total cost of diagnostic tests in the open and in the standardized groups respectively. However, in our study, we did not include the cost of fluorescein angiography unlike Lee et al. In our study, more tests were ordered, especially more laboratory investigations, but the cost per patient in the standardized group was still lower.

A similar cross-sectional survey (comprising 4 anterior uveitis scenarios) was conducted among practicing ophthalmologists, fellows, and residents in Canada [15]. Similar to Lee’s et al. results a wide range of tests were ordered by the 498 responders and many of the tests were found to be of low diagnostic yield by the authors. Cost minimization and sensitivity analyses showed that applying the guidelines may lead to cost savings when compared with current practice patterns across the different scenarios that were evaluated (p<0.01) [15]. The maximal additional cost was observed with non-granulomatous anterior uveitis investigation (minimal additional cost of $75/patient). The additional cost was $40 for granulomatous uveitis whereas it was $36 when sarcoidosis was suspected. In Noble’s study, the cost of granulomatous and non-granulomatous anterior uveitis was $121 and $101, respectively. In our study, the cost of diagnostic tests for granulomatous and non-granulomatous anterior uveitis was higher (167.33€ and 158.59€, respectively).

The main limit of this study is that we assumed that the two diagnostic strategies were equivalent with respect to the clinical effectiveness of diagnosing uveitis. However, in the ULISSE study, an etiological diagnosis was established in 54% of cases in the open group and 50% in the standardized group (non-significant, p = 0.20). The difference between both strategies (standardized minus open) was -4.9% [95% CI (-12.5%; 2.6%)]. The comparison of both strategies remained inconclusive because the standardized strategy was neither inferior nor non-inferior to the open one [16]. Furthermore, as developed previously [16], in the standardized group, 25.6% of the patients were lost to follow-up, limiting the power of our study as well as its validity. Most of these patients were young, active men with acute anterior uveitis, a condition that may decrease follow-up adherence.

In addition, the cost-effectiveness ratio could not be evaluated in this study, since the only costs assessed were those of medical procedures. Investigators did not report the costs related to hospitalizations, treatments and work stoppages. It is therefore not possible to calculate this ratio representing a difference in overall costs of care and efficiency.

In conclusion, this ancillary analysis of the ULISSE multicentre, randomized study evaluating the cost-consequence of a standardized strategy vs. an open one shows that significantly fewer investigations were performed in the standardized group resulting in a significantly lower mean cost per patient. Such an approach can therefore lead to important cost savings.
